# Are Australian immigrants at a risk of being physically inactive?

**DOI:** 10.1186/1479-5868-8-53

**Published:** 2011-06-01

**Authors:** Jayantha Dassanayake, Shyamali C Dharmage, Lyle Gurrin, Vijaya Sundararajan, Warren R Payne

**Affiliations:** 1 Center for Molecular, Environment, Genetic and Analytical Epidemiology, The University of Melbourne, Australia; 2 Department of Medicine, Southern Clinical School, Monash University, Australia; 3 Institute of Sport, Exercise and Active Living, Victoria University, Australia; 4 Faculty of Nursing, University of Alberta, Canada

## Abstract

**Background:**

We examined whether physical activity risk differed between migrant sub-groups and the Australian-born population.

**Methods:**

Data were drawn from the Australian National Health Survey (2001) and each resident's country of birth was classified into one of 13 regions. Data were gathered on each resident's physical activity level in the fortnight preceding the survey. Multivariable logistic regression, adjusted for potential confounders examined the risk of physical inactivity of participants from each of the 13 regions compared to the Australian-born population.

**Results:**

There was a greater prevalence of physical inactivity for female immigrants from most regions compared to male immigrants from a like region. Immigrants from South East Asia (OR 2.04% 95% CI 1.63, 2.56), Other Asia (OR 1.53 95% CI 1.10, 2.13), Other Oceania (1.81 95% CI 1.11, 2.95), the Middle East (OR 1.42 95% CI 0.97, 2.06 [note: border line significance]) and Southern & Eastern Europe are at a significantly higher risk of being physically inactive compared to those born in Australian. In contrast, immigrants from New Zealand (OR 0.77 95% CI 0.62, 0.94), the UK & Ireland (OR 0.82 95% CI 0.73, 0.92), and other Africa (OR 0.69 95% CI 0.51, 0.94) are at a significantly lower risk of being physically inactive compared to the Australian born population.

**Conclusion:**

Future research identifying potential barriers and facilitators to participation in physical activity will inform culturally sensitive physical activity programs that aim to encourage members of specific regional ethnic sub-groups to undertake physical activity.

## Background

Cardiovascular disease (CVD) mortality rate has decreased in developed countries [1,2]. However, this decrease varies according to ethnicity, sex and socio economic status [2,3]. Therefore, in countries with a diverse ethnic population, there is a need to understand the risk factors for CVD among specific ethnic communities to better inform interventions aimed at reducing the risk of population-wide CVD. An important component of many ethnic communities is the immigrant population.

Physical inactivity is a risk factor for CVD [1,4-6] and many studies have shown that immigrant adults have higher physical inactivity levels than adults from the host Western population [7,8]. Further, physical activity is an important risk factor for diabetes and Australian studies have found higher rates of diabetes in some immigrant groups [9,10].

Studies have not explored the physical inactivity levels of a comprehensive range of first generation immigrant communities in relation to a single Western host country except for the Australian study by Bennett et al. [11]. Bennett and colleagues reported that male immigrants from Scotland and Ireland, Southern Europe, Middle East and South East Asia were at greater risk of being physically inactive in their leisure time compared to an Australian-born population. Similarly, female immigrants to Australia from Southern Europe, Middle East, South East Asia and Other Asia were at greater risk of being physically inactive in their leisure time compared to the Australian born female population. They reported that no immigrant population group was significantly more physically active than the Australian-born population.

Notwithstanding its comprehensive nature, the study by Bennett et al. [11] was conducted on data collected from 1980-1989 and the pattern of immigration throughout the world and to Australia specifically, in the intervening period has altered substantially [11]. Further, the level of physical activity (PA) completed by adult Australians and many other western populations has decreased significantly since 1989 [12]. The approach used by Bennett and colleagues to measure physical activity participation was to classify individuals as being physically inactive only when they reported no leisure time physical activity (ie sedentary). In recent times the concept of a threshold value below which one is considered to have insufficient physical activity to incur a health benefit by a population has been introduced [13]. One such threshold is the accumulation of 1600 kcal of leisure time physical activity in a two week period [13]. Therefore, the current literature does not enable an understanding to be gained of the relative risk of physical inactivity in a large range of current first generation immigrants after they move to a Western culture using more contemporary approaches to the measurement of leisure time physical activity.

An ideal setting for such a study is Australia, where immigrants comprise 23% of the adult community and are drawn from more than 200 countries representing all regions of the world [14]. The purpose of this study was to determine whether there was a significant association between the level of physical inactivity and a comprehensive range of adult first generation immigrant sub groups in Australia.

## Methods

The Australian National Health Survey (NHS) 2001 was a cross-sectional self-reported, randomly sampled, population-based survey conducted by the Australian Bureau of Statistics (ABS) in 2001 [13]. Data were collected using face-to-face interviews in the respondent's language of choice by trained interviewers. A de-identified dataset from the NHS 2001 survey was provided to the researchers. The data set was reduced from 21,000 to 19,175 inhabitants of private dwellings after eliminating unoccupied dwellings and dwellings where respondents were aged less than 15 y.

A total of 4,956 first generation migrants were selected from the data base. Country of birth was used to classify the immigrant sub-groups into 13 regions according to the Australian Standard Classification of Countries [15]. The regional classification included: born in Australia, New Zealand, Other Oceania, UK and Ireland, Other North Western Europe, Southern and Eastern Europe, North Africa, the Middle East, Other Africa, South East Asia, Other Asia, Americas, and Others [15]. The number of first generation migrants identified from the database for each region and their gender are outlined in Table 1.

**Table 1:** Prevalence of physical inactivity by gender, NHS 2001

	Male			Female			
**Region of origin**	**Number**	**Inactive %**	**95% CI**	**Number**	**Inactive %**	**95% CI**	P-value*
Australia	6460	61.67	0.60, 0.62	7759	72.37	0.71, 0.73	0.00
New Zealand	203	58.13	0.51, 0.64	215	65.58	0.58, 0.71	0.10
Other Oceania	43	69.77	0.56, 0.83	59	86.44	0.77, 0.95	0.04
UK & Ireland	740	60.95	0.57, 0.64	806	69.11	0.65, 0.72	0.00
Other North Western Europe	205	65.37	0.58, 0.71	196	64.80	0.58, 0.71	0.90
Southern & Eastern Europe	460	71.74	0.67, 0.75	503	78.73	0.75, 0.82	0.01
North Africa	37	64.86	0.49, 0.80	20	75.00	0.56, 0.93	0.43
Middle East	82	63.41	0.52, 0.73	65	86.15	0.77, 0.94	0.00
Other Africa	85	52.94	0.42, 0.63	86	60.47	0.50, 0.70	0.32
Americas	194	75.77	0.69, 0.81	304	83.55	0.79, 0.87	0.03
South East Asia	98	72.45	0.63, 0.81	96	76.04	0.67, 0.84	0.56
Other Asia	90	66.67	0.56, 0.76	110	75.45	0.67, 0.83	0.17
Others	119	72.27	0.64, 0.80	140	79.29	0.72, 0.86	0.18

*P-values for differences between males and females in each population subgroups.

The data included a self reported estimate of the level of PA undertaken during leisure time for sport, recreation, or health and fitness purposes during the two weeks prior to the interview [13]. Data on the frequency of and total amount of time spent in the various forms of PA were also collected. The proportion of respondents who did or did not achieve the level of leisure time PA required to derive a health benefit was calculated using scores derived from the estimated energy expenditure and calculated metabolic equivalents (METs) for each form of PA [12]. The threshold for classification being as physically activity was an aggregate leisure-time energy expenditure of 1600 Kcal over the two weeks data collection period.

The prevalence of physical inactivity among the 13 immigrant sub-groups was compared to the Australian-born population. The odds ratios and 95% confidence intervals were calculated using the categorical outcome of physical inactivity/activity by the country of birth, sex and age compared to the Australian-born population. Multivariate analysis was performed using a logistic regression model while adjusting for possible confounders of age, sex, personal income, educational qualification, occupation, and year of arrival in Australia. The statistical analysis was performed using STATA 9.0 (p < 0.05). The study was approved by the University Human Research Ethics Committee.

## Results

Table 1 describes the prevalence of physical inactivity according to region of origin and sex. All female groups, except Other North Western Europe were inactive compared to the corresponding male population. This difference was significant for those born in Australia, UK and Ireland, Southern and Eastern Europe, Other Oceania, Middle East and the Americas.

Table 2 outlines the multivariate odds ratios for the association between region of origin and physical inactivity adjusted for age, sex, personal income, educational qualification and occupation.

**Table 2:** *Multivariate odd ratios for prevalence of physical inactivity, NHS 2001

Region of origin	Odd ratios	95% CI	P-value
Australia	1.00		
New Zealand	0.77	0.62, 0.94	0.01
Other Oceania	1.81	1.11, 2.95	0.01
UK & Ireland	0.82	0.73, 0.92	0.00
Other North Western Europe	0.83	0.67, 1.03	0.10
Southern & Eastern Europe	1.25	1.07, 1.46	0.00
North Africa	1.07	0.60, 1.88	0.81
Middle East	1.42	0.97, 2.06	0.05
Other Africa	0.69	0.51, 0.94	0.02
South East Asia	2.04	1.63, 2.56	0.00
Other Asia	1.53	1.10, 2.13	0.01
Americas	1.29	0.94, 1.76	0.10
Other	1.73	1.29, 2.32	0.00

*The association between country of birth and physical inactivity was adjusted for age, sex personal income, education qualification, occupation, and year of arrival perform multivariate odds ratios.

Using the above findings the physical inactivity levels of immigrants in relation to the host Western country were grouped into three bands.

Band 1. Regions that were significantly less physically inactive than the Australian born population i.e. New Zealand, UK & Ireland, and Other Africa

Band 2. Regions those were significantly more physically inactive than the Australian born population i.e. Other Oceania, Southern & Eastern Europe, Middle East, South East Asia, and Other Asia. The Middle East region was of boarder line significance (p = 0.05) and for the purposes of this exploratory paper has been considered to be significantly different.

Band 3. Regions not significantly different in prevalence of physicalinactivity compared to the Australian born population, i.e. Other North andWestern Europe, North Africa, and Americas

## Discussion

The present study is a novel examination of the level of physical inactivity among immigrants to a single western country - Australia, from the world's regions. Irrespective of the region of origin, the prevalence of physical inactivity of females was either significantly greater or not significantly different to that of the males from the same region of origin. Specifically, regions where the prevalence of physical inactivity in females was significantly greater than males included Australia, Other Oceania, UK and Ireland, Southern & Eastern Europe, Middle East and the Americas. These findings are in keeping with those previously published [16] and it has been suggested that the time constrains arising from a women's role in domestic and child raising activities may be contributing factors to them being less active than their male counterparts [17]. It is important to note that as the interaction between gender and region of birth for physical inactivity was not significant, comparisons between region of birth and physical inactivity for each gender have not been discussed.

No previous studies have been located which report that first generation migrants have significantly less physical inactivity than the host population (Band 1). In the current study, those in Band 1 were immigrants that were culturally closest to the majority of Australians (e.g. those born in the UK & Ireland). These data are important as the literature has generally focused upon migrant groups at risk of being physically inactive in comparison to the host population. The existence of Band 1 contrasts to the findings of Bennett et al. [11] for the period 1980-1989 as they reported no instances where the immigrant group was less physically inactive than the host Australian population.

In contrast to the lack of studies with which to compare the Band 1 grouping, a large number of studies support the Band 2 grouping. Dawson et al. [18] and Bennett [11] reported that male and female immigrants from Southern and Eastern Europe to Sweden and Australia, respectively, were at a higher risk of being physically inactive in their leisure time than the members of the host population. Our findings on South East Asian and Other Asian immigrants were also consistent with those found in the Australian study by Bennett [11] where both male and female immigrants from South East Asia and females from Other Asia were found to be at a high risk of physical inactivity. Immigrants from South East Asia have also been shown to be at high risk of being physically inactive compared to the Canadian-born population [7]. At a country level, similar associations were found for Vietnamese women compared to the Swedish born population [11], Indian immigrants in the United Kingdom [19,8], and Sri Lankan immigrants in Canada [7]. However, Mexican born immigrants to the USA were at higher risk of being physically inactive compared to USA born counterparts [20]. These data contrast to the current study where immigrants to Australia from the Americas were not reported to have significantly different physical activity levels in comparison to the Australian-born population. However, it is acknowledged that regionally-based data may mask country specific data such as that presented by Sundquist [20].

The location of immigrants from the Middle East in Band 2 was consistent with the previous Australian research in this area; although it should be noted that the p-value was 0.05. Bennett found that both male and female immigrants from the Middle East were at a higher risk of being physically inactive compared to the Australian-born population. The findings of this study were consistent with recent evidence that has suggested that women who migrated from the Middle East [7,11] had high levels of physical inactivity in their leisure time. These findings, largely among women of the Muslim faith, were related to their inability to speak the language of the host country, the effect of religious beliefs on participation in leisure time PA, avoidance of mixed sex activities, and fear of going alone to activities [21]^.^

The regions in Band 3 included Other North and Western Europe, North Africa, and Americas. While acknowledging the methodological differences in the calculation of physical inactivity between the current paper and that of Bennett [11], the only region reported by Bennett [11] where the prevalence of physical inactivity was in agreement with that observed in the current study was Other North and Western Europe. Bennett [11] reported that the prevalence of physical inactivity for migrants from New Zealand and England and Wales and Scotland and Ireland (women only) was no different to that observed in the host Australian population. In the current study, migrants from these regions now had significantly less physical inactivity than the host Australian population.

It should be noted that, in general, grouping countries together to single regional group may have masked some true country specific effects and therefore, this is a limitation of these data. In particular, most regions are comprised of a number of ethnic and cultural groups who may have different and potentially counteracting levels of high and low levels of PA.

The difference in immigrant PA behaviour compared to the Australian-born population might be due to cultural affinity with leisure time PA. This was potentially the case with migrants to Australia from Southern Europe as immigrants from this region did not express any positive attitudes or interests towards leisure time physical activity [22]. In addition, leisure time physical activity was reported as an unpopular activity among both men and women from Asian immigrant groups living in Australia [11].

A second potential explanation for the different physical activity behaviours of migrants and the Australian-born population might be the level of acculturation to the host culture. The acculturation hypothesis states that the level of immigrant physical activity is strongly associated with ability to speak the host language and to engage with the host culture [23]. Evidence from the United States, [23] the UK [21] and Australia [11] supports this hypothesis. For example, first generation Latino immigrants to the USA with higher levels of the English language were more physically active than those with lower levels of English [15]. This hypothesis might also explain why data in our study showed that immigrants from UK & Ireland, and New Zealand had higher levels of physical activity, as they may have better English language skills and be more acculturated to Australian society and its physical activity structure than those born in non-English speaking countries.

A final factor that may affect migrant physical activity participation is socio-economic status. Immigrants who have lived for longer periods in their host countries have an increased level of leisure time physical activity and this is related to their level of disposable income [15]. Further, the direct association between disposable income, available leisure time and physical activity level may also contribute to the finding that individuals from Western regions have increased levels of physical activity [11].

As mentioned previously, the current study specifically builds upon that reported in 1993 by Bennett and colleagues. In addition to the temporal difference between the collection periods for the two data sets and the ever changing regional pattern of immigration, the current study contributes to our knowledge of migrant health and physical inactivity as it explored physical activity independently as an exposure variable while adjusting for a large range of possible confounders.

The major strength of this study is the high quality of the data gathered. Nevertheless, issues surrounding the measurement of physical activity in epidemiology studies have been widely debated [13]. In the present study, a PA score derived from METs was used. This method has been used in previous large international cross-sectional community surveys [5]. However, the NHS, 2001 did not measure occupational physical activity and this is likely to have caused some under reporting of PA. This is particularly relevant in the measurement of PA in migrants as it has been demonstrated that some migrant groups undertake a relatively large amount of occupationally-related PA [24]. However, it is also equally important to mention that the cultural biasness due to different interpretations of the terms 'physical activity' and 'exercise' between cultural groups might be a another limitation of this study [10].

In addition, the data used from the 2001 NHS is self-reported and therefore, recall bias may exist. Finally, the use of regional classifications, as mentioned previously, may mask individual country-specific and cultural differences and the small numbers in some regional immigrants groups may have affected the precision of estimate and in some instances large differences were not statically significant.

## Conclusion

Female immigrants from Other Oceania, UK and Ireland, Southern and Eastern Europe, Middle East and Other Asia were at a significantly higher risk of being physically inactive than their males counterparts. The prevalence of physical inactivity in regional migrant groups differed substantially and can be grouped into three band: Band 1, significantly less physical inactivity than the host country; Band 2, significantly more physical inactivity than the host country; and Band 3 no significant difference from the host country. These data are substantially different to those published in the landmark multi-regional study by Bennett et al using data from the 1980s. It is suggested that future studies should conduct research into factors that influence physical inactivity levels among these high risk immigrant groups and determine culturally accepted solutions to promote physical activity.

## Practical Implications

• Community physical activity interventions need to acknowledge the varying physical inactivity patterns of ?individual migrant groups.

• Health related physical activity promotion programs should focus upon those migrant groups at greatest risk of physical inactivity.

## Competing interests

The authors declare that they have no competing interests. No external financial support received.

## Authors' contributions

JD carried out study design, data analysis, and writing of the manuscript. SD provided input the study design, analytical design and write-up of the manuscript. LG contributed to the design of the study, advised on database structure, managed data and performed the statistical analysis. VS provided input into the analytical design and write up of the manuscript. WP provided input into the study design, interpretation of data and writing of the manuscript. All authors read and approved the final manuscript.
